# The ambivalence of losing weight after bariatric surgery

**DOI:** 10.3402/qhw.v9.22876

**Published:** 2014-01-29

**Authors:** Christine Warholm, Aud Marie Øien, Målfrid Råheim

**Affiliations:** 1 Department of Global Public Health and Primary Care, University of Bergen, Bergen, Norway; 2 Department of Social Sciences, University College of Sogn and Fjordane, Sogndal, Norway

**Keywords:** Obesity surgery, weight loss, women, lived body, lived experience, phenomenology

## Abstract

This study is grounded in a phenomenological lifeworld perspective. It aims at providing rich descriptions of lived experience of the process of losing weight after obesity surgery. Two women participated in in-depth interviews four times each during the first postoperative year. Based on the women’s experiences, a meaning structure—the ambivalence of losing weight after obesity surgery—was identified across the women’s processes of change. This consisted of five core themes: *movement and activity*—freedom but new demands and old restraints; *eating habits and digestion*—the complexity of change; *appearance*—smaller, but looser; *social relations*—stability and change; and *being oneself*—vulnerability and self-assurance. These core themes changed over time in terms of dominance. The experience of ambivalence is discussed according to a phenomenological perspective of the body as lived experience.

The prevalence of overweight and obesity in the Western world is increasing, and it has been characterized as a global trend. Its economic repercussions are huge, and the World Health Organization (WHO) considers obesity to be one of our greatest public health concerns (WHO, 2013). The trend has also reached Norway (Reas, Nygård, Svensson, Sørensen, & Sandanger, 2007; Ulset, Undheim, & Malterud, 2007). In 2003, one out of every five Norwegians was, by definition, obese (Ulset et al., 2007). WHO (2006, 2013) defines overweight according to Body Mass Index (BMI, kg/m^2^), and a BMI above 40, or above 35 if accompanied by obesity-related diseases (diabetes, cardiovascular diseases, musculoskeletal disorders, and increased risk of cancer), represents severe obesity. Persons with severe obesity have reported decreased quality of life compared to persons with normal weight (Karlsson, Taft, Rydén, Sjöström, & Sullivan, 2007). They face prejudice and stigma on a daily basis due to their physical appearance and body size, and prejudice among health personnel (Malterud & Tonstad, 2009; Pain & Wiles, 2006; Salant & Santry, 2006). Hence, the consequences of severe obesity are manifold.

It is international consensus that bariatric surgery is the final option when all other attempts at weight loss have failed (Buchwald, 2005), and it is the most rapidly growing treatment for severe obesity. In Norway, the number of bariatric surgery procedures is increasing: 1900 in 2008 (Kristinsson, 2008), and 2000 in 2010 (Hofsøe et al., 2011). This includes both surgeries done in public hospitals as well as those performed in private clinics. In private clinics, patients pay for the surgery themselves. Different surgical procedures are used; these are categorized into restrictive procedures, where the size of the stomach is reduced, and malabsorption procedures, where the small intestine is diverted and connected differently within the bowel. Often, these two methods are combined (Kristinsson, 2008). The effect of bariatric surgery is defined according to the amount of weight loss, changes in diseases related to overweight, mortality, quality of life, and postsurgical complications (Aasheim, Mala, Søvik, Kristinsson, & Bøhmer, 2007). Several studies show that bariatric surgery leads to substantial weight reduction, especially during the first postoperative year (Norwegian Regional Health Authorities (NRHA), 2007); improvement in quality of life; and increase in psychosocial function (Aasprang et al., 2008; Karlsson et al., 2007; Nickel, Loew, & Bachler, 2007; Våge, Solhaug, Viste, Bergsholm, & Wahl, 2003; van Hout, Boekestein, Fortuin, Pelle, & van Heck, 2006). Research from the Swedish Obese Subjects Study (SOS study) showed that even after 15 years, obesity surgery was associated with reduced mortality (Sjöström et al., 2007). However, the surgery itself is also a risk. A meta-analysis showed that postoperative mortality after gastric bypass was 1%, and that 10–20% of patients experienced several complications and side effects, such as reflux and vomiting, also called dumping (Deitel, 2008; Maggard et al., 2005), as well as foul-smelling stools and diarrhea (NRHA, 2007). Considerable weight regain is also reported and is considered a treatment failure (Fried et al., 2007). Patients also reported problems with excess skin due to the weight loss (Spector, Levine, & Karp, 2006), for instance as a degrading factor that can influence the individual’s body image (Song et al., 2006). These studies rest on survey-based data. A deeper understanding of these patients’ experience of psychosocial function and embodied change is called for (van Hout et al., 2006).

Qualitative studies exploring the first-person perspective after bariatric surgery are growing. Based on patients’ stories, Bocchieri, Meana, and Fisher (2002) described bariatric surgery as a rebirth and transformation for the better, but not without tensions related to vast changes experienced by the participants. The complexity of the experience of food and eating is underscored in more interview studies, and is related to weight control or wish for weight control, and/or health-related habits and practices (Engström & Forsberg, 2011; Groven, Engelsrud, & Råheim, 2012; Murray, 2005; Natvik, Gjengedal, & Råheim, 2013; Ogden et al., 2006). Perception of control is suggested to be an essential aspect of patients’ postsurgery experiences of their own bodies (Jensen et al., 2013). Living with excess skin and scars after bariatric surgery is shown to create ambivalence and discomfort, experienced as a constant reminder of the impossibility of fully escaping the previously large body (Groven, Råheim, & Engelsrud, 2013). In a study by Groven, Råheim, and Engelsrud (2010), it is also shown how the participants experienced their lives to become dramatically worsened following bariatric surgery. With the exception of the SOS study, most studies that investigate quality of life and psychosocial function after surgery are investigated in cross-sectional designs. This is also the case for most qualitative interview studies exploring life after obesity surgery. An exception is Engström and Forsberg’s study (2011), where patients were interviewed prior to and 2 years after surgery. In order to provide patients with realistic expectations for the surgery and a better understanding of the changes after the operation, there is still a need for more knowledge about lived experiences of changing bodies after bariatric surgery through longitudinal designs. The purpose of this study was to gain a deeper understanding of the lived experience concerning the vast bodily and other changes that the severely obese go through during the first postoperative year. The research questions were: How do patients experience their own bodies after obesity surgery, and in what ways do these experiences influence daily living? How does the lived experience of their own bodies change from immediately after surgery to 1 year after?

## Methodology

Investigating lived experience calls for a lifeworld perspective, grounded in phenomenology. “Lifeworld” means the world of daily experience, the world in which we live and to which we ascribe meaning. Spiegelberg (1982, p. 144) termed it “the world of lived experience.” When exploring the participants’ experiences, the in-depth lifeworld interview was found to be relevant (Kvale, 1996). To cultivate a phenomenological attitude was a key issue during the interviews and the analysis. That meant cultivating an attitude of openness and wonder, as well as putting pre-understandings at risk, as for instance described by Dahlberg, Dahlberg, and Nyström (2008). Because the objective was to capture lived experience of change over time, the design was longitudinal. Interviewing several times made it possible to gain insight into how the process of change was experienced, with special regard to the experience of one’s own body.

## Method

### Participants

To be included in the study, patients had to meet the following criteria: They must have undergone the surgical procedure called biliopancreatic diversion with duodenal switch (BPD/DS, a malabsorption procedure), be between the ages of 30 and 50 years, be of Norwegian ethnicity, and speak Norwegian as a native language. Participants were recruited from a public hospital in western Norway during the period of October 2007 to May 2008. A nurse outside the research team, who worked at the hospital’s surgical ward with these patients in particular, aided the recruitment. The nurse informed potential candidates at the pre-surgical conversation, which was 1 week before their surgical treatment. Three participants gave their written informed consent, which was sent directly to the first author; the first two of these were included. The third participant was not included because the consent was received 9 months after the interview phase started. The researchers considered that inclusion this late in the study could hamper a thorough analysis because of restricted time. The participants were women, one in her 30s and single, the second in her 40s and married; neither had children. Both worked full-time, but they were on sick leave when the study started. They had both undergone BPD/DS surgery after living with severe obesity since their late childhood.

### Conducting the interviews

Participants were interviewed separately, at 3, 6, 9, and 12 months during the first postoperative year. The first author conducted all the interviews in an office at the university. Each lasted for 60–80 min and was tape recorded. An interview guide was used for each interview, containing keywords and themes without standardized questions. Depending on what was told in the first interview, the interview guide was modified before the next interview in line with the flexibility of a qualitative design (Malterud, 1993, 2012). This gave the researcher an opportunity to go deeper into the experiences and themes that were most important for the participant at that particular time in the first postsurgical year. This made it possible to determine whether an important aspect of their experience had changed or not, which was crucial in order to detect the participant’s processes. The starting point focused on how they lived their lives at the time of the interview, especially how they experienced their own bodies after the surgical intervention. The participants were encouraged to give detailed descriptions of experiences, situations, and events. They were also asked to reflect upon the meanings of these experiences, which was inspired by the phenomenological approach as well as the life-form interview (Haavind, 2000). For example, from interview 3: “In the previous interview you talked about how you had noticed that it was easier for you to define your feelings compared to before the surgery. How do you experience this now?”. Follow-up questions were: “Can you give an example?” and “What do you think about this being easier now?”.

### Analysis

The tape recordings were transcribed by the first author after each interview. Further analysis was carried out by the first author, based on thorough discussion between the three authors. Systematic Text Condensation was used (Malterud, 2003, 2012), containing four steps. The research team followed these steps, but made adjustments and changes. The team did not analyze the interviews across participants from step 1 onward, as recommended by Malterud (2003, 2012). In this study, it was important to obtain the process of change within each case. However, cross-case analysis was conducted in step 4. In the last step, the team realized that the research material was rich enough to transcend to a more generalized level than Malterud’s analysis (2003, 2012) incorporates. Additionally, the researchers were influenced by van Manen’s (1997) underscoring of writing as an integrated part of the analysis, including his thoughts on evocative writing.

#### Step 1: Get a total impression

During the first step, the focus is to gain an initial overview of the entire material by carefully reading through the transcribed interview texts two to three times, and listening to the tape recordings. The first author read through the first transcribed interview and listened to the tape recording for each participant separately. This was done as preparation for the next interview, which is a deviation from Malterud’s method (2003, 2012) but in line with the longitudinal design. However, when all the transcribed interviews were available, the interviews from each participant were read and reread separately by all authors to gain a total impression of each process. The first author summarized the preliminary themes identified in each process, keeping in mind the lived experience of the body. Already at this stage, significant meanings in preliminary themes seemed to overlap in both processes.

#### Step 2: Identify meaning units

The second step consisted of the identification of meaning units in the texts, keeping in mind the themes found in step 1. All statements were numbered consecutively, relating the text elements to context. Then, interviews were reread, and elements containing relevant information were identified. Subsequently, meaning units were taken out of context and sorted thematically in matrices. The meaning units needed further refinement to demonstrate the nuances of each specific theme. Codes were therefore chosen to describe each nuance within each theme. For an example, the theme *social relations* had codes named work, family & friends, and meeting the surrounding world. During this process, some themes were renamed and new themes were discovered. Codes were redefined accordingly. The matrices were detailed and included all four interviews. This made it possible to gain an overview of the meanings of each coding group. Themes and codes were then visualized in figures and provisional working models. These illustrated the interrelations between the themes and codes, as well as how the process changed for each participant during the first postoperative year.

#### Step 3: Abstraction of meaning units

In this step, the researchers reviewed the meaning units and reformulated their content of meaning, based on the participant’s experiences. The starting point was the coding groups. Meaning units in each coding group were rewritten and redefined, producing artificial quotations that illustrated the meaning of each coding group in a more general statement, as recommended by Malterud (2003, 2012).

#### Step 4: Identifying core themes and a general structure

The last step was to recapitulate the meaning of all the coded elements and reformulate their core meanings by re-describing them. Specifically, this consisted of re-contextualizing by integrating the meaning units into consistent descriptions at a higher level of abstraction, based on the artificial quotations in step 3. At this stage, the team was able to identify main themes in each woman’s process. Based on these, the researchers were also able to identify five core themes across the longitudinal processes. Condensed descriptions of these themes were formulated, and verbatim extracts and shorter quotations from the transcribed material were included. During this step, the descriptions were verified against the matrices from step 2 to establish that the described meaning was within its context. At this stage, the researchers repeatedly discussed and dwelled on what they termed “movements between opposites” in the participants’ stories: losing control and feeling in control, feeling self-assured but still being afraid of losing oneself, enjoying the freedom and joy of being invisible in the crowd but being sad and frustrated about re-discovering how degrading being an obese body is in society, and so on. The team went back to the condensed and detailed descriptions of the core themes, and explored if these kinds of movements were significant, which was found to be the case. Accordingly, during this last part of the analysis, the team was able to identify a meaning structure of the experiences of transforming bodies across the processes and core themes: the ambivalence of losing weight after obesity surgery.

Further theoretical interpretation of the women’s experiences according to a phenomenological perspective of the body as lived experience is highlighted in the discussion.

### Ethical considerations and procedures

The study was approved by the Regional Committee for Medical Research Ethics and the Norwegian Data Inspectorate. A written consent was signed by both participants. The interviewer was aware of sensitive issues at play in the conversations, and was cautious not to stir up strong emotions too much. The opportunity to meet over time, however, gave the participants the chance to re-think and re-discover. Both participants expressed how being part of the study was a positive experience. They pointed at taking part as enabling them to give words to the changes they went through, which seemed to contribute to acknowledging own experiences.

## Lived experience of transforming bodies

The presentation will first describe significant meanings of lived experience of the processes of change after bariatric surgery identified in the meaning structure, which is the ambivalence of losing weight after obesity surgery. It will also demonstrate how significant meanings changed for both women. Some core themes were more dominant in the beginning of the first year, and others at the end. When presenting the core themes, firstly a condensed description based on both women’s experiences will be introduced. Then the presentation will concentrate on one of the women, who is called Nina, giving a more detailed description of lived experience during the first postoperative year.

### The ambivalence of losing weight after obesity surgery

During the first year after surgery, dramatic and rapid bodily changes took place, which changed the women’s lives profoundly. During the process, their stories were characterized by ambivalence, feelings of joy and sadness, increased freedom and new restrictions, vulnerability and self-assurance, and hopes and worries for the future. The lived experience of the gains and losses associated with a rapidly changing body was the turning point, and it was intertwined in all core themes. Lived experiences of *movement and activity* and *eating habits and digestion* dominated their stories in the first interviews, while experiences of *appearance*, *social relations*, and *being oneself* were in the foreground in the last interviews.

**Figure 1:**
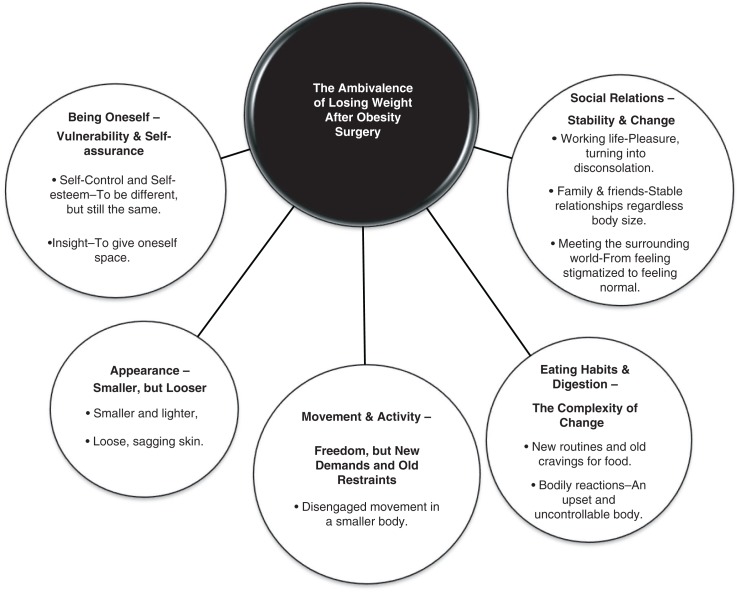
The ambivalence of losing weight after obesity surgery. The figure illustrates the general structure with its five core themes and related subthemes.

**Figure 2:**
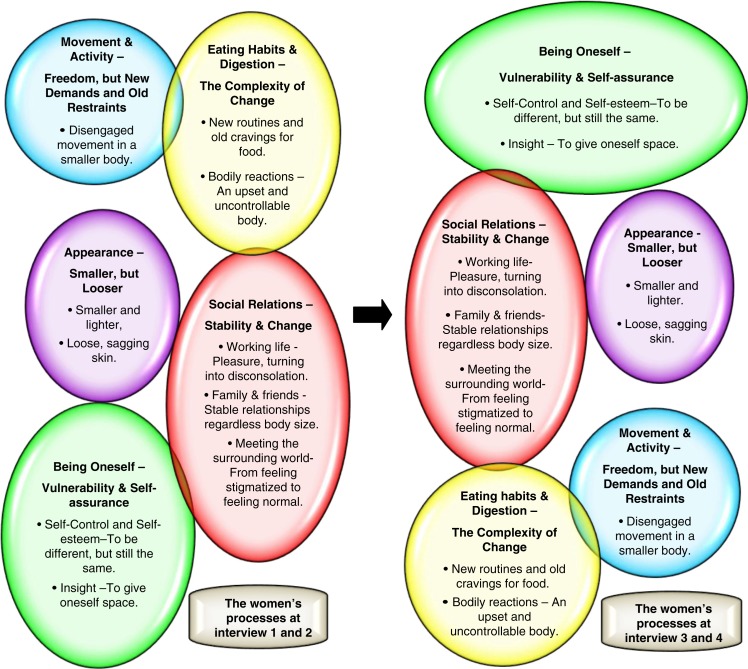
The process of losing weight after obesity surgery. The figure illustrate how the different themes of lived experience changed for the women during the first year after surgery. Each bubble represents a core theme. The left side represents the first two interviews, and the right the last two interviews. The figure shows how the themes changed in dominance during the year by altering the positions of the bubbles. Bubbles on the top indicate the themes that played a dominant role for the women, while the ones at the bottom were less dominant.

The core themes, with their respective subthemes, are illustrated in Figure 1. The women’s processes according to the dominance of the core themes throughout the first year after surgery are shown in Figure 2.

### Movement and activity—freedom, but new demands and old restraints

Both women described how the weight loss drastically changed their mobility, even quite soon after the surgery. To be able to move without great effort in smaller bodies was a significant new experience that meant new and long-hoped-for opportunities in life. Nevertheless, as time went by, less attention was paid to this new freedom, which gradually became the norm. Despite increased mobility and the resulting increase in participation in various activities, the women expressed feelings of guilt throughout the year for not exercising as much as they felt they should. They tried hard to adapt to a new lifestyle.

#### Disengaged movement in a smaller body

Nina experienced profound changes in her mobility, and she found it wonderful to be able to participate in activities she earlier could not be a part of, and to do everyday tasks that had previously been difficult or impossible. In the first interview, she expressed a newborn freedom:

It feels really good! It really does! Being able to sit down on the sofa and pull my legs up completely is something I previously could never do! I no longer have to be frightened of standing too much at work. Now I can actually do my work without feeling pain, it feels wonderful!

Her body no longer prevented her from moving around, which was an incredible feeling and something she had longed for. These experiences were prominent in the first two interviews. During the two final interviews, she described her mobility as more of an everyday experience, and she did not reflect that much on the fact that her days now included more activities. Even though her level of daily activities increased considerably during the process, she had difficulty starting regular exercise routines, and she described this as a problem. She had negative experiences with physical exercise from the past, remembering being stared at when doing exercise because she looked strange when moving about. This was hard to forget, and she claimed she would never attend a fitness center ever again: “I have never fitted in in places like that!” At the same time, she felt guilty for not exercising as much as she was told to. New demands were hampered by old restraints. Nevertheless, in the last interview she still hoped that exercise would become less difficult in the future.

### Eating habits and digestion—the complexity of change

From day 1 after the surgery, the women had to deal with food in a different manner. Unfamiliar reactions from a dramatically changed digestive system, and resisting their old eating habits, were daily challenges. They knew this was a process they simply had to go through. Gradually, they learned how often and how much to eat, but not without pain from testing their bodies’ new limits. During the two last interviews, a new control of eating habits seemed to be established. However, a troublesome digestive system due to the surgery affected their well-being and social situation for the entire postoperative year.

#### New routines and old cravings for food

In the first interview, Nina was concerned with how she had to change her eating habits, and how this caused unfamiliar and frustrating situations. After surgery, she had to eat more often than before, but very little at a time, and she also had to make choices about which food to eat. Previously, she had not been accustomed to planning her day around meals, and she felt she did not manage it the way she should. She constantly had to deal with her old and strained feelings related to eating, realizing that these still made an impact on her: “I want to be able to eat three slices of pizza instead of only a half!” Although she realized that the amount of food she used to eat was a big problem, she still missed the feeling of comfort that food had given her. Nevertheless, in the third interview Nina had acquired better routines for meals and eating habits. She had gained new know-how about her altered body’s digestive functions, and had a renewed ability to listen to the reactions of her own body. Even though old cravings for food occasionally threw her off-balance, she felt more in control in the last interview.

#### Bodily reactions—an upset and uncontrollable body

Nina’s digestion gave her a lot of trouble throughout the first year after surgery. She expressed how foul-smelling stools and diarrhea were hampering issues, leading to embarrassing and uncomfortable situations when she was among other people. Another discomfort was vomiting, especially when she ate too much or too fast: “I was scared of getting hungry, so I threw in some food, and then: vomiting!” She also felt ill when she did not eat on time. The first two interviews were characterized by Nina’s experiences of living with an upset and uncontrollable body. In the third interview, she was more able to cope with her changed body regarding food and eating habits and bodily discomforts. Nevertheless, she still had difficulties in the final interview. However, she referred to these as “bagatelles” compared to the gains after surgery.

### Appearance—smaller, but looser

Excitement and delight followed when the participants noticed how the weight loss changed their looks for the better. The experience of not being stared at outside the safe haven of home was something they truly cherished, as well as the feeling that they looked not just normal but also good. However, weight loss also brought about increasing worries when their external looks also changed for the worse. The constantly increasing excess skin became a matter of significant concern during the final two interviews. The women were anxious about what opportunities there were to correct the skin, which in the last interview represented uncertainty.

#### Smaller and lighter

Nina described how the weight loss changed her looks and how it influenced the way she presented herself to others. In the second interview, she said, “I used to feel safe with my jacket on, because it covered a bit. Now I feel I can leave the jacket at home and still feel good!” In the third interview, she expressed that daring to reveal her body and, in this sense, be more visible in typical summer clothing gave her a good feeling. At the same time, the considerable changes also made her wistful. When putting away her old and now too-big clothes, she said, “It’s like putting away pieces of myself.” These clothes symbolized old feelings and memories. Nevertheless, packing them away gave her a feeling of satisfaction, as the primary meaning of the act was that her body had become smaller. She was ambivalent.

#### Loose, sagging skin

The more weight Nina lost, the more her skin sagged. Already in the first interview, she worried about what the future would bring and was certain that she soon would be in need of plastic surgery. She spent hours thinking about how she would look afterward, and said: “It’s not only about getting thin; one has to look normal as well!” Despite being more familiar with her body’s new appearance, Nina acknowledged in the third interview that she felt really bad about the loose, sagging skin. In the final interview, she was relieved when her surgeon said that plastic surgeries were within reach. Finally, she could see the end of the whole process. She craved “normality.”

### Social relations—stability and change

Stability and change in relation to others were woven into the women’s stories throughout the entire process. This included participation at work and the value of support from family and friends. It also encompassed how the women approached the world around them, which gave unexpected experiences. Their stories described meeting a gentler world when they were lighter and thinner, a world where they were not stigmatized. The fact that they were treated differently due to their new appearances was difficult to accept. At the same time, they were relieved to be able to walk around unnoticed in public. Ambivalent feelings about the way they were met by the surrounding world were present.

#### Working life—pleasure turning into disconsolation

Nina returned to work shortly after the surgery, but she found it especially difficult to cope with her body’s new demands for food and rest. Still, she gave the job priority. The job gave her a feeling of being respected for her abilities and knowledge. Therefore, suddenly losing her job halfway in the process marred her pleasure. She had difficulty picturing herself out in the job market. In the third interview, she said, “Losing my job is a fly in the ointment for me. It’s quite difficult to deal with right now.” She was still influenced by her previous job-hunting experiences, where she often found that she was judged by her appearance and not her qualifications. Nevertheless, she claimed that this time it would be different. She was both excited and hesitant.

#### Family and friends—stable relationships regardless of body size

Support from family and friends was of great significance for Nina. The fact that those closest to her could now see that life had finally turned out well for her was of special importance, and she was deeply grateful that nothing was profoundly changed in her relationships with her family. They treated her like they always had, regardless of her weight. In the third interview, she said, “My mom says, ‘I’ve got a new girl in so many ways, but still, it’s you!’”

#### Meeting the surrounding world—from feeling stigmatized to feeling normal

During the first two interviews, Nina shared experiences about how she was used to being met by the world as an overweight person. She was accustomed to brutal and inconsiderate surroundings outside the safe haven of family and friends. She felt stigmatized and was faced with comments about how she herself was to blame for leading her own body into misery. She said, “It was as if people could say whatever they wanted to me.” The world showed a lack of respect toward her, and she concluded that this was not the case for people whose body size was normal. Then she suddenly found herself to be one of the crowd. Facing a gentler and more inclusive world was a valuable experience that changed her perspective. At the same time, this experience provoked and hurt her, because she felt she was still the same person. In the final interview, she expressed the difficulty of letting go of her feelings connected to the years of stigmatization. Even though Nina felt bitterness, the most important thing seemed to be the new feeling of freedom:

As a matter of fact, the days are totally different! I’m just so pleased to be able to walk around without being bothered, you know. The feeling of being invisible! This is something I value most of all!

### Being oneself—vulnerability and self-assurance

This core theme was prominent in the two last interviews. The changes the women went through generated new feelings for their bodies, which influenced the way they looked upon themselves. Despite this, old and suppressed feelings emerged during this process, which they found difficult to sort out and deal with. Their stories were full of conflicts and a sense of losing control of the changes they underwent. At the same time, feelings of a new self-assurance were manifested along with the changes, as well as the emergence of a new self-insight. In the final interview, they felt stronger and had new hopes and wishes for the future.

#### Self-control and self-esteem—to be different, but still the same

Throughout the process, Nina constantly tried to keep track of what was happening to her body, and to be in charge of her own feelings. She struggled not to lose herself. She expressed being scared that getting thinner would make her feel like a “better” person than the one she used to be. In the first interview, she said, “I do think I will be more self-assured, but I don’t think I want to change personality, because I am so focused that this cannot happen!” At the same time, the process made her realize how the weight loss changed her in more subtle ways. In the second interview, she was surprised when she said: “Now I am more able to put words on what is wrong, painful or what is bothering me compared to before. It’s easier for me to express myself!” Furthermore, as time went by, she faced the fact that the rapid and continuous weight loss was difficult to relate to, and in the third interview she stated, “I haven’t been part of the transformation. I mean, my mind hasn’t absorbed the changes my body has gone through.” She was of course aware of being thinner, but in some cases she still behaved as if she was overweight, which was confusing. In the last interview, however, she described a newfound ability to relax and participate more fully, and explained how the bodily upheavals played a smaller part in her life. She was finally able to let go of the control she had fought so hard to keep, and felt she could master her life in new ways. Ambivalent feelings were still involved, though, including a special frustration about the fact that the size of her body had such an impact on the feelings about herself. She claimed: “You see; I really used to like the person I was before. So even if ‘she’ had a difficult time, I was really fond of her too!” Nevertheless, she was content that her personality became more visible with her new self-assurance, and had hopes for the future.

#### Insight—to give oneself space

During the process, Nina gradually gained new insight into herself and why things had been as they were before the surgery. Realizing how her weight used to stop her from being fully herself, she understood how her feelings and self-expression had been inhibited by her large body. In the first interview she said, “I know I can, but it hasn’t been easy to express myself in a body like that.” The overweight had caused her to renounce herself, and in a way she felt as if the person she was had been hidden and even forgotten inside her body. Years of self-suppression had marked her, and after the surgery her hidden feelings could be released. This was a new freedom, but also frightening. In the three final interviews, she spent time dwelling on her decision to go through with the surgery, which had taken her years. She realized why the decision had been so difficult: “This was my defeat, because I couldn’t do it myself. It was embarrassing, not being able to lose weight on my own. That’s why it was so hard to decide.” Looking back, it surprised her that it took her so long to get help. She claimed that the operation was first and foremost “a savior” for her health, and an instrument for taking part in a greater variety of life. However, she also felt bitterness because she had to go through this dramatic process in the first place. In the final interview, her ambivalent feelings were obvious: “It’s still difficult, the fact that I did this just as much for others as for my own sake.” Memories of not being accepted by society were still painful.

## Discussion

The researchers will discuss three aspects of essential meanings of the ambivalence of losing weight after obesity surgery: *The ambivalence of old and new body habits and practices*; *The embodied self*—*Weight loss as a means of losing as opposed to finding oneself;* and *The obese person’s perception of the world versus the world’s perception of the obese person*. To deepen the understanding, we will draw on Merleau-Ponty’s phenomenology of the body, as his perspective adds substantially to the understanding of bodily change. We will also draw on earlier research. Finally, methodological reflections will be considered.

### The ambivalence of old and new body habits and practices

In the first two interviews, we saw that the women’s processes were characterized by bodily changes that caused joyous feelings of new freedom of movement and a transformed appearance. At the same time, these first months were a struggle. The women strove to adapt to the body’s new demands for food, and they had to deal with an upset body that was difficult to control. As time went by, however, new abilities and practices were acquired, especially in terms of eating habits. In the last interview, the experience of transforming bodies was more normalized. However, they struggled to “keep up with” their changing body.

According to Merleau-Ponty’s (1962) thinking, our daily life’s habits and skills are anchored in the body. The acquisition of new habits is a rearrangement and a renewal of the corporeal schema, and an expression of our ability to change our existence (Merleau-Ponty, 1962). The corporeal schema is “an open system of an infinite number of equivalent positions” (Merleau-Ponty, 1962, p. 163) that enables us to experience our body and act as bodies in a variety of situations. It encompasses two layers: the layer of the habit body, which includes embodied know-how and dispositions, and the body at this moment, which acts and perceives based on our present bodily based understanding. With this as a starting point, it can be argued that the changes the women went through represented a disintegration of the body’s two layers, which for them were the more familiar and obese habit body and the constantly changing body in the moment. The disintegration created a distance from their own body and world, an experience of unfamiliarity, as the body changed so drastically over a short period of time. They were, especially in the early phase of the process, left with the feeling of living in a body out of control. As we have seen, for Nina it took time to reunite the known, obese body and her experiences with the new, smaller, and changing body, a process that was still going on in the last interview. Hence, from a phenomenological point of view, it was the disintegration she initially felt that had made her develop new habits as well as new ways of acting toward her surroundings.

Other studies investigating patients’ experiences after obesity surgery highlight their efforts to regain lost control after surgery. Engström and Forsberg (2011) followed patients for 2 years after obesity surgery and found that the process of change involved a fundamental struggle to create a sustainable control, especially in regard to eating habits and weight. In addition, Throsby (2008) argued that following obesity surgery, patients face an ongoing disciplining of the “self,” and she underscored that patients have to relearn how to eat with a surgically modified stomach, which also means handling the sociality of eating in new ways. Several studies support this understanding of the complexity of changing eating habits and the importance of control. They describe learning to cope and relating to bodily changes as long-lasting and difficult processes (Bocchieri et al., 2002; Groven et al., 2012; Jensen et al., 2013; Natvik et al., 2013; Ogden et al., 2006).

### The embodied self—weight loss as a means to losing as opposed to finding oneself

The dimension of losing as opposed to finding oneself was deeply enmeshed in the women’s processes of change. As we have seen in Nina’s example, it was initially of great importance to her not to change her personality. Nevertheless, her process showed how this was a challenge, and how she had conflicting feelings toward the changes she underwent. The rapid speed of the weight loss made the alienation the women felt toward their own bodies quite dramatic. In the two last interviews their experiences were characterized by how the weight loss had changed their perspective on themselves, which also meant ambivalent feelings. From the researchers’ point of view, the process showed how the weight loss enabled the women to take a meta-perspective on former unsorted feelings and recapture their inner self.

According to Merleau-Ponty (1962), our access to the world is through our lived bodies. With this in mind, both women’s processes were grounded in their lived life as obese persons. Merleau-Ponty (1962) described how serious illness and bodily pain influence the way we as body subjects perceive our lifeworld. He spoke of illness and childhood as ways of being in the world (Merleau-Ponty, 1962). Inspired by Merleau-Ponty’s thinking, Leder (1990) stated that when the body is healthy and functions well, we take the lived body for granted. The lived body then slips away from our attention in a kind of absent presence or disappearance, as a prerequisite for us being fully absorbed in our projects in the lifeworld. However, when illness or disability strikes, the body itself becomes the center of thematic attention—the body is “dysappearing,” as Leder (1990) terms it, in our experience. Notably, the women’s stories accounted for how their bodies changed for the better and for the worse, accompanied by, in Leder’s (1990) terminology, new kinds of disappearance as well as dysappearance. Their stories accounted for how their obese bodies had prevented them from fully taking part in life. In many ways, their weight was a decisive factor in how they had lived their lives and perceived themselves. As shown in Nina’s case, not only had she been hampered by overweight in regard to mobility, which influenced a variety of activities, but not being able to control her weight also gave her feelings of failure and shame. The large body was what was seen by others, and it kept her from expressing herself intellectually and emotionally to others. These experiences were deeply rooted in Nina and were thus fundamental for how she perceived the first year after surgery. In the last interview, she was able to reflect on what being overweight meant in relation to the way she had perceived and expressed herself. According to Merleau-Ponty (1962) a change in the lived body also involves a change in our lifeworld. From a phenomenological perspective, it is suggested that losing a significant amount of weight became a radical change in these women’s worlds.

Studies emphasize patients’ feelings of alienation toward themselves and their surroundings as significant, especially in the first period after the surgery (Bocchieri et al., 2002; Ogden et al., 2006). Patients in Engström and Forsberg’s study (2011) told about improvement in self-image and self-confidence 1 year after surgery, and that they were able to look back and realize that it had been the obesity that had caused their bad self-esteem before surgery. Natvik et al. (2013) revealed that re-discovering oneself was part of the long-lasting processes after bariatric surgery. Additionally, Throsby (2008) highlighted how the participants in her study viewed the surgery as a means to enable a more disciplined body. She stated that they were “rescued from obesity and restored to a more authentic, socially legitimized, disciplined self” (Throsby, 2008, p. 129). Our study shows that losing weight after obesity surgery indeed rearranged these two women’s worlds.

### The obese person’s perception of the world versus the world’s perception of the obese person

The women in our study described an amazing feeling of invisibility when among others in public spaces, which was highly valued. After losing weight, the women learned to face a gentler world, which made them feel more normal and self-assured. Consequently, the weight loss not only changed their perception of themselves but also changed their view of the world and the world’s perception of them. Nevertheless, memories of feeling stigmatized by society were deeply rooted. They had a hard time accepting the narrow-mindedness of society.

The ambivalence in the women’s stories in this respect reveals integrating an external view of their own bodies, which was anchored in the external gaze of others before the surgery, as well as resisting it. As we have seen, by looking at herself from outside, Nina’s body became an object that was not socially acceptable, an object of social devaluation. In the aftermath, Nina looked back at her experiences and could realize the narrow-mindedness of society. In line with Zahavi (2004), Nina’s ambivalence can be highlighted by the fact that our existence is not only based on our own perception of ourselves but also intertwined in how others perceive us. According to Merleau-Ponty (1962), the subject and the world are inseparable, and the body is an incarnated subject that embodies experience and culturally mediated meanings.

The sociologist Erving Goffman (1963) explains how society establishes means to categorize people into “normal” and “abnormal,” defining the term *stigma* as a deeply discrediting attribute that involves feelings of shame. Carr and Friedman (2005) argue that obese people are stigmatized in accordance with the dimensions described by Goffman (1963): namely, the stigma is related to the obese body itself, which is defined as abnormal, and the obese person’s character, which links obesity to morality. In their study, they found that obese people felt stigmatized in situations related to work, health, and daily life in general (Carr & Friedman, 2005). During the process of weight loss, Nina re-discovered social norms about being severely obese. She was reluctant to identify with social norms about “normal” body size that say that *not* being severely obese also means being, morally speaking, a better person. Puhl and Brownell (2001) emphasized that several studies have argued that obese people are one of the very few remaining groups in society that still is deemed legitimate to stigmatize. Murray (2005, p. 155) showed how society views obese people as being out of control, weak, and unwilling to change, and stated that this view establishes the obese body as “a failed body project.” Gaining weight after bariatric surgery is described to create a double failure: being severe obese in the first place, and then failing to keep control of the weight even after bariatric surgery (Groven et al., 2010). Knutsen, Terragni, and Foss (2013) interviewed morbidly obese participants who attended a course before surgery and self-help groups afterward, both focusing on lifestyle. The researchers found that dimensions of control and credibility framed the respondents’ identity work. They suggest that treatment programs aiming at empowering these patients leave the patients trapped within the ambivalence between freedom and control. Again, body size, health, illness, and lifestyle are phenomena steeped in discourses about control and morality, which our study also points to.

### Trustworthiness and transferability—methodological considerations

Establishing trustworthiness is the way in which a researcher manages to produce findings that are worth paying attention to (Lincoln & Guba, 1985). Several criteria are highlighted in the literature (Lincoln & Guba, 1985; Malterud, 2001; Stige, Malterud, & Midtgarden, 2009; Whittemore, Chase, & Mandle, 2001). Reflexivity on the part of the researcher is drawn to the fore, for example by identifying the researchers’ preconceptions in regard to the study, as issued by Malterud (2001). In this study, the motivation arose from the first author’s clinical encounters as a physiotherapist with a patient who went through obesity surgery. The rehabilitation process made a deep impact due to the vast bodily changes. The first author was interested in learning more about how these changes were experienced, this being a relatively new field in Norway. The two other authors, also physiotherapists and researchers, had a broad knowledge within the field of physiotherapy, but not with patients going through obesity surgery.

It is important to apply the most optimal method, which is dependent on the research questions asked (Whittemore et al., 2001). We aimed for rich descriptions of lived experience of transforming bodies. In-depth interviews are relevant when exploring lived experience (Kvale, 1996), and a longitudinal design ensured a focus on the process of change during the first year after the surgery. It can be argued, however, that the small sample size in this study is a weakness, as the findings only represent experiences from two processes after obesity surgery. At the same time, the small sample ensured the possibility to go deeply into each woman’s process. Rich descriptions are not first and foremost based on the number of participants, but on delving more deeply into lived experience (Dahlberg et al., 2008). In addition, a small sample size ensures the possibility of a thorough analysis (Malterud, 2001).

The first author conducted all the interviews, but all authors participated in planning the study, analyzing the interviews, and writing the article, which strengthens the internal validity, as well as member checks (Lincoln & Guba, 1985; Malterud, 2001; Whittemore et al., 2001).

In terms of transferability, with the help of a stepwise, phenomenologically inspired analysis, we were able to identify a meaning structure across the women’s processes of change, which consisted of five core themes. In our discussion, we emphasized rich descriptions in the presentation of the material and further theoretical interpretation of essential meanings according to the phenomenology of the body. By presenting one of the women’s stories in more detail, new insight into the complexity of the process that takes place after obesity surgery is highlighted. All together, we have aimed for both particularity and knowledge at a higher level of generality. In this way, we also focused on pragmatic validity (Kvale, 1996), which parallels the question of how the processes described could facilitate change (Stige et al., 2009). If found relevant by healthcare workers who encounter these patients, the findings can stimulate alterations in the way the healthcare system—including physiotherapists—attends to these patients during the first year after surgery. In addition, knowledge about the process of change is relevant, both for patients who plan to go through obesity surgery and for their relatives. Our study has provided new insight into such processes, but more knowledge is needed. We hope to facilitate further research about the complexity of bodily change after obesity surgery.

## Conclusion

Compared to previous research, our study gives a more detailed description and discussion of the fundamental changes in the lifeworlds of embodied selves that occur when losing weight during the first postoperative year after obesity surgery. The women’s stories in our study also reveal how fundamental aspects change during the first year in terms of dominance in the women’s lived experience. Together with previous qualitative research, the study points to the need for a closer follow-up from health personnel in the postoperative period. In line with Galvin, Todres, and Dahlberg’s notions about “lifeworld-led” care (2013), we argue that lived experience should be acknowledged as an important source of knowledge in the rehabilitation of the obesity surgical patient. That also implies an existential view of the human being, her suffering, and her well-being, and an existential partnership between patient and healthcare worker.
